# Experiential Thinking in Creationism—A Textual Analysis

**DOI:** 10.1371/journal.pone.0118314

**Published:** 2015-03-03

**Authors:** Petteri Nieminen, Esko Ryökäs, Anne-Mari Mustonen

**Affiliations:** 1 University of Eastern Finland, Faculty of Health Sciences, School of Medicine, Department of Biomedicine/Anatomy, P.O. Box 1627, FI-70211, Kuopio, Finland; 2 University of Eastern Finland, Philosophical Faculty, School of Theology, P.O. Box 111, FI-80101, Joensuu, Finland; 3 University of Eastern Finland, Faculty of Science and Forestry, Department of Biology, P.O. Box 111, FI-80101, Joensuu, Finland; University of Sheffield, UNITED KINGDOM

## Abstract

Creationism is a religiously motivated worldview in denial of biological evolution that has been very resistant to change. We performed a textual analysis by examining creationist and pro-evolutionary texts for aspects of “experiential thinking”, a cognitive process different from scientific thought. We observed characteristics of experiential thinking as follows: testimonials (present in 100% of sampled creationist texts), such as quotations, were a major form of proof. Confirmation bias (100% of sampled texts) was represented by ignoring or dismissing information that would contradict the creationist hypothesis. Scientifically irrelevant or flawed information was re-interpreted as relevant for the falsification of evolution (75–90% of sampled texts). Evolutionary theory was associated to moral issues by demonizing scientists and linking evolutionary theory to atrocities (63–93% of sampled texts). Pro-evolutionary rebuttals of creationist claims also contained testimonials (93% of sampled texts) and referred to moral implications (80% of sampled texts) but displayed lower prevalences of stereotypical thinking (47% of sampled texts), confirmation bias (27% of sampled texts) and pseudodiagnostics (7% of sampled texts). The aspects of experiential thinking could also be interpreted as argumentative fallacies. Testimonials lead, for instance, to ad hominem and appeals to authorities. Confirmation bias and simplification of data give rise to hasty generalizations and false dilemmas. Moral issues lead to guilt by association and appeals to consequences. Experiential thinking and fallacies can contribute to false beliefs and the persistence of the claims. We propose that science educators would benefit from the systematic analysis of experiential thinking patterns and fallacies in creationist texts and pro-evolutionary rebuttals in order to concentrate on scientific misconceptions instead of the scientifically irrelevant aspects of the creationist—evolutionist debate.

## Introduction

The creationist–evolutionist debate derives from the 1800’s but its modern form became active in the 1960–70’s [1–3]. Young-earth creationism (YEC) [1–2] does not accept the geological age of the earth but holds on to a special creation approximately 6000 years ago. Old-earth creationism (OEC) [1–2] accepts the geological sciences but denies the gradual change of organisms. YEC and OEC proponents theorize, instead, that animals appeared as “individually created kinds” (felids, canids, bovids, *etc*.), between which no evolution is possible. Intelligent design (ID) appeared in the late 1980’s and its ideas ultimately derive from the argument from design [2, 4–5]. ID proponents state that complex biochemical and morphological structures or behavioral patterns could not have evolved without supernatural intervention [2, 4].

McGrath [4] classifies the dialogue between science and religion into four types. “Conflict” is exemplified by the heated discussion on evolution and represented by YEC, OEC and ID. “Independence” assumes science and religion to be non-overlapping spheres of reality [4, 6]. “Dialogue” and “integration” include increasing harmonization of scientific and religious worldviews [4]. The last three models are represented by theistic evolution (TE; “creationism” only in the broadest sense). TE proposes that a deity has used cosmology and natural laws as tools in creation *via* evolution [7]. It is accepted by major Christian denominations [8–11]. TE advocates have attempted to integrate evolutionary theory with spirituality. Instead of using scientific data to prove the existence of a deity, McGrath [4] interprets religion as a way to make sense of scientific observations.

Examining the cognitive methods the debaters utilize to acquire evidence for one’s claims could yield interesting data. In fact, patterns of thought similar to creationism occur regarding persistent beliefs in alternative healing methods despite of ample counter-evidence [12]. This seems to be caused by a phenomenon psychologists call “experiential thinking”. This is a process that practically everyone employs in everyday problem solving. It is evolutionarily old and can lead to rapid processing and decision-making and be highly beneficial in adapting to the environment. As seen below, the processes employed to gain evidence in experiential thinking (testimonials, confirmation bias, *etc*.) have apparent similarities to argumentative fallacies, such as appeals to emotional aspects instead of the actual content of a claim, which can lead to false beliefs and their enforcement [13–14].

Creationist texts representing the “conflict” model often use arguments derived from re-interpretation of scientific data. Generally, creationists are interested in science and do not necessarily question scientific observations *per se* but disagree with mainstream biology on their interpretation [15]. Claims by creationists regarding their interpretation of natural sciences have been thoroughly analyzed and discussed [16] and they are not the main focus of the present study. Creationist texts also include material that is irrelevant to disproving evolution [17]. These passages contain argumentative fallacies that include demonization and portrayal of evolutionary biologists as unreliable or unqualified (direct *ad hominem* fallacy [18]). Creationists also claim that scientists themselves would not be convinced about evolution (*tu quoque* [17]). Evolutionary theory is associated to hideous consequences, such as Nazism and mass murders (guilt by association, *ad consequentiam* and slippery slope fallacies [19–20]). Instead of scientific evidence, creationist theory can be justified by using appeals to authority [19] and complicated data can be distorted into only two alternatives with false dilemmas or simplified with hasty generalizations [21]. While these types of fallacies are not *per se* relevant when discussing scientific evidence, they are potentially effective when persuading an audience [13]. Thus, it may not be adequate only to recognize and dismiss fallacious arguments but their context should also be examined and discussed, and it would be useful for science educators to assess why the creationist–evolutionist debate relies partly on scientifically irrelevant and fallacious arguments. Wilkins [22] has suggested that creationist thought depends on the “sort of exposure” a learner has regarding scientific issues. Education can have a crucial role here but teaching is sometimes conducted only as a presentation of facts without adequate explanations about the underlying principles. Furthermore, the basic commitments (or “biases”) of a learner can be incompatible with scientific data. Thus, a learner may find some parts of science unacceptable and abandon these parts based on pre-existing biases.

We hypothesized that some of the characteristics of creationism that appear to be nonscientific and resistant to change could be at least partly explained by the utilization of experiential thinking. In addition, as both experiential thinking and fallacious argumentation can induce and fix false beliefs [12–13], there could be a relationship between the thinking patterns and fallacies. The specific aims of the present analysis were to assess systematically if aspects of experiential thinking can be observed in creationist texts and in rebuttals written by natural scientists and to investigate if there is a possible connection between experiential thinking and the observed argumentative fallacies. We focused on the discussion belonging to the “conflict” model of the interaction between science and religion [4], as it remains relevant for a large part of the general public. For instance, 40% of the U.S. population advocate the teaching of YEC and/or ID in schools [23]. We were able to confirm our hypotheses as the aspects of experiential thinking were very clear and the argumentation analysis showed that these aspects could be interpreted as fallacies. Based on this, we suggest a scheme for science educators and participants of the creationist–evolutionist debate on how to address creationist claims by systematic analysis.

## Methods

Creationist writings representing YEC, OEC and ID were selected based on their visibility, impact and citations in creationist and evolutionist texts and social media (Table 1). TE texts were excluded as TE accepts biological evolution and does not attempt to falsify theories of natural sciences [2, 4]. To compare the situation to non-English speaking populations, we also analyzed highly-cited Finnish creationist writings. The texts were classified as YEC, OEC or ID, but there was a lot of overlap between ID and OEC, which is indicated by ID/OEC. We also analyzed the overall context of the texts, *i*.*e*., if they concentrated on, for instance, personal aspects of evolutionary scientists or alleged social consequences of accepting evolution or if they also discussed the “scientific” claims for creationism. Based on previous studies and reviews [12, 24–30], we analyzed aspects of experiential thinking as follows:

1. Concrete information with personal experience as a principal tool for assessment of data. Instead of scientific evidence, testimonials and narratives are employed.
2. Confirmation bias is prevalent in hypothesis testing. This leads to underutilization or dismissal of negative instances or null information and seeking of information that is consistent with existing beliefs. Contradictory data are ignored or judged unreliable.
3. Pseudodiagnosticity, in which information is regarded as relevant even if it is not. This includes disregard for base rate information and/or sample size.
4. Complex and threatening information is re-organized into a controllable form. Concepts used in experiential thinking are holistic, concrete and emotional. There is a tendency for broad generalization and stereotypical thinking.
5. Morally neutral issues are given moral significance and there can be magical beliefs (things acting on each other at a distance due to sympathy or supernatural means). Opinions based on experiential thinking are resistant to change and not easily transformed by logical evidence.

**Table 1:** Sources of principal sample material.

Institution/Author	Format	Type	Source/Publisher
Answers in Genesis	Online articles	YEC	https://www.answersingenesis.org/
Creation Ministries International	*• Creation Magazine*	YEC	http://creation.com/
	*• Journal of Creation*		
Creation Research Society	*• Creation Matters*	YEC	https://www.creationresearch.org/
	*• CRS Quarterly*		
Institute for Creation Research	Online articles	YEC	http://www.icr.org/
Intelligent Design and Evolution Awareness (IDEA) Center	Online articles	ID/OEC	http://www.ideacenter.org/
Intelligent Design network	Online articles	ID	http://www.intelligentdesignnetwork.org/index.htm
UK Apologetics	Online articles	YEC	http://www.ukapologetics.net/
Behe MJ	*The edge of evolution*: *The search for the limits of Darwinism* [108]	ID	Free Press
Johnson PE	*• Darwin on trial* [36]	ID/OEC	http://www.talebooks.com
	*• Reason in the balance*: *The case against naturalism in science*, *law & education* [44]		InterVarsity Press
Puolimatka T (in Finnish)	*• Faith*, *science and evolution* [19]	ID/OEC	Uusi Tie
	*• A test for openness in science discussion* [74]		
Reinikainen P (in Finnish)	*• The forgotten Genesis* [47]	YEC	Uusi Tie
	*• The enigma of the dinosaurs and the Bible* [105]		Kuva ja Sana
	*• Darwin or intelligent design* [21]		Uusi Tie
Davis P, Kenyon DH	*Of pandas and people*: *The central question of biological origins* [48]	ID	Haughton
Morris HM	*The remarkable birth of planet earth* [32]	YEC	Bethany Fellowship

YEC = young-earth creationism, OEC = old-earth creationism, ID = intelligent design.

During the analysis, particular oft-repeated creationist claims were noted to fit several of the above-mentioned aspects of experiential thinking. Due to this, some claims and creationist references appear on multiple occasions in the Results section. After organizing the findings according to the distinctive features of experiential thinking, the examined creationist claims were compared to argumentative fallacies [14]. This allowed assessing the potential relationships between the experiential thought patterns and fallacies. Finally, we propose a scheme for educators and debaters on how to address creationist claims not only based on their “scientific” content but also on the analyses of thinking and argumentation.

To yield comparative data on the possible presence of similar thinking patterns in pro-evolutionary refutations, we also analyzed a sample of these texts, but actual peer-reviewed evolutionary papers in scientific journals with no connection to the creationist–evolutionist debate were not included. The prevalences of different aspects of experiential thinking were recorded for the cited sample material of the three text types (YEC, ID/OEC and pro-evolutionary; the reference [31] was excluded as it only contains a short citation). Multiple occurrences of a particular aspect within a single text were not calculated separately. The prevalences were analyzed with the χ^2^ test or, if the test criteria were not met, with the Fisher’s exact test (SPSS *v*19 software package, IBM, Armonk, NY, USA). The p value < 0.05 was considered statistically significant. The results are presented as % of texts with at least one occurrence of a particular aspect of experiential thinking. In addition, examples of these aspects are presented to enable other scholars to recognize them. An example of analysis and the raw data are available as Supporting information (S1 and S2 Tables).

## Results

### Testimonials as evidence in creationism

**Fig 1:**
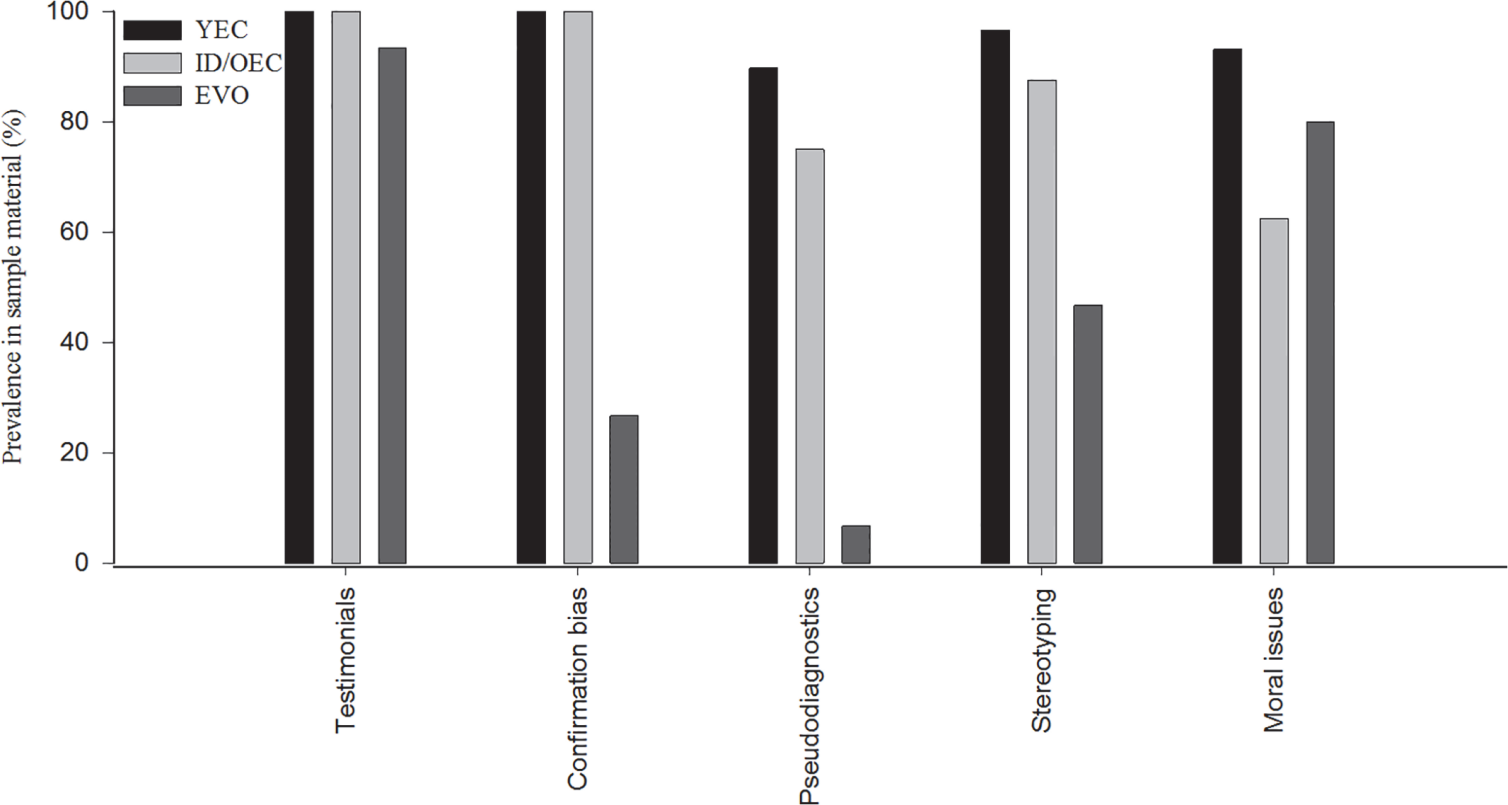
Prevalences (%) of selected aspects of experiential thinking in the sampled material representing young-earth creationism (YEC; n = 29), intelligent design/old-earth creationism (ID/OEC; n = 8) and pro-evolutionary texts (EVO; n = 15). “Testimonials” include personal testimonies, quotes, appeals to authorities, *etc*. “Confirmation bias” represents ignoring or dismissing contradictory data and alternative hypotheses. “Pseudodiagnostics” entails giving high relevance to misinterpreted or irrelevant issues. “Stereotyping” includes dichotomies and generalizations and “moral issues” refer to scientifically irrelevant discussion of moral implications to prove or disprove a claim. * = Difference between the text types (χ^2^-test, Fisher’s exact test, p < 0.001).

Creationist texts revealed a habit of justifying scientific claims with testimonials, as all sampled YEC and ID/OEC texts contained them (prevalence 100%; Fig. 1). We could further classify these as follows:

- Testimonials of the author(s): personal testimony supporting creationism or refuting evolutionary theory including appeals to incredulity.
- Testimonials (often citations) of authoritative figures supporting creationism or disproving evolution. These can also include “unknown authorities”.
- Testimonials of evolutionary scientists used as evidence against evolutionary theory. These include (out-of-context) quotes of alleged fatal flaws in evolutionary theory.
- Testimonials on the personal characteristics of evolutionary proponents or on the demonic nature of evolutionary theory *per se*.
- Selected scientific results (often taken out of their original context) as testimonials either for creationism or against evolution.

In the first case, the writer simply uses one’s personal authority to either support creationism or denounce evolutionary theory (Table 2) with simple statements as follows: “[…] I personally have become thoroughly convinced that the Biblical record […] gives the only scientific and satisfying account of the origin of all things” [32]. The second type of testimonial is that of authoritative figures supporting creationism or disproving evolution: “There are hundreds, perhaps thousands, of scientists today who once were evolutionists but have become creationists in recent years” [19, 21, 32–33]. Similarly, past scientists are summoned to provide testimony that Christian faith is a prerequisite for scientific research of high quality [19, 21] although the issue is controversial [34]. A repeatedly occurring authoritative figure as a witness is Popper and his statement (later renounced [35]) that evolutionary theory would not be falsifiable [36]. Quotes of evolutionary scientists are utilized to give testimony about alleged fatal problems in evolutionary theory. These can be presented as “involuntary admissions” of evolution being based on dishonest or biased research. The most common citations deal with alleged scarcity of transitional forms in the fossil record and structures that evolutionary theorists supposedly admit not being able to evolve naturally (Table 3).

**Table 2:** Examples testimonials as proof in creationist writings.

Type	Portrayal or citation	Type	Source
Personal testimony for creationism	“Experience confirms this; a plan always requires a designer and information always indicates intelligence. Everybody of us knows this.”	YEC	[100]
	“Thankfully, most people are not hopelessly deceived. Polls in America show that the majority believes in creation, and many more want it taught.”	YEC	[39]
	“I am personally a former atheist. My conviction was based on evolutionary theory […]”	YEC	[47]
Personal testimony against evolution	“[…] I delved into the matter. I was able to tackle some of the arguments of Dawkins […] conclusions of Stephen Jay Gould […] of Carl Sagan […] I considered carbon dating […] By the time I completely finished with all of this […] I felt it was time to put evolutionism to bed […]”	YEC	[75]
	“When I want to know how a fish can become a man, I am not enlightened by being told that the organisms that leave the most offspring are the ones that leave the most offspring.”	ID/OEC	[36]
	“[…] I do not think that the pattern of nature […] ‘proves’ common ancestry.”	ID/OEC	[44]
	“The idea that a complex structure or system can somehow be formed by chance is a persistent delusion accepted by evolutionists […] But this idea is absurd.”	YEC	[32]
Character witness against evolutionists	[Regarding Darwin] Plagiarization, desire to avoid persecution, cowardice, guilt, racism, doubtful qualifications	YEC	[18, 75]
	“Many evolutionists I have met have something in their own past that has turned them away from ‘religion’ […] A bitter hatred of God and Biblical truth developed […]”	YEC	[39]
	“Mass murderer Jeffrey Dahmer, for example, lived his life believing evolution was true history […]”	YEC	[101]
	“The fact is that evolutionists believe in evolution because they *want* to.”	YEC	[102]
Character witness against evolutionary theory	“[…] killing of so many millions of people, let alone the onslaught on defenceless unborn babies […] is totally consistent with evolutionary teaching […]”	YEC	[42]
	“So if evolutionary teaching destroys the faith of Christians, then it is anti-Christ in nature. And if it is a lie, its true origin is from the father of lies [Satan].”	YEC	[103]
	“Evolution is the root of atheism, of communism, nazism, behaviorism, racism, economic imperialism, militarism, libertinism, anarchism, and all manner of anti-Christian systems of belief and practice.”	YEC	[32]
Citations of evolutionary proponents as character witness against evolution	“Biological arguments for racism may have been common before 1850, but they increased by orders of magnitude following the acceptance of evolutionary theory.” [originally in 104]	YEC	[31]

YEC = young-earth creationism, OEC = old-earth creationism, ID = intelligent design.

**Table 3:** Examples of oft-repeated citations of evolutionary scientists as evidence against evolution.

Citation	Creationist sources	Original source	Actual context or omitted parts of the citation
“The extreme rarity of transitional forms in the fossil record persists as the trade secret of paleontology.”	[19, 36, 48, 74]	[106]	Gould defends his idea of punctuated equilibrium.
“We paleontologists have said that the history of life supports that interpretation [gradual change], all the while really knowing that it does not.”	[19, 36, 74]	[107]	Eldredge defends the idea of evolution not being steady but sometimes quite rapid (punctuated equilibrium).
“If it could be demonstrated that any complex organ existed, which could not possibly have been formed by numerous, successive, slight modifications, my theory would absolutely break down.”	[19, 36, 105, 108]	[70]	“[…] But I can find out no such case.”
“Why then is not every geological formation and every stratum full of such intermediate links? Geology assuredly does not reveal any such finely graduated organic chain […]”	[48, 74]	[70]	Darwin refers to the fossil record not being perfect.
“To suppose that the eye, with all its inimitable contrivances for adjusting the focus to different distances, for admitting different amounts of light, and for the correction of spherical and chromatic aberration, could have been formed by natural selection, seems, I freely confess, absurd in the highest possible degree.”	[47]	[70]	”Yet reason tells me, that if numerous gradations from a perfect and complex eye to one very imperfect and simple, each grade being useful to its possessor, can be shown to exist […] then the difficulty of believing that a perfect and complex eye could be formed by natural selection, though insuperable by our imagination, can hardly be considered real.”
“[…] not a single valid example is known of phyletic [gradual] transition from one genus to another.”	[48, 109]	[110]	Transitions available between taxa at higher levels (order, phylum).

Testimonials regarding the personal characteristics of evolutionary theorists were widespread (Table 2). While speculations on the moral character of scientists can be based on real character flaws, it should be recalled that the validity of a theory rests on evidence and not on the person. Creationists referred to evolutionary proponents as racist, dishonest, cowardly, gullible, psychotic, sadistic, unqualified and—in the case of Darwin—of sickly family background, because “family inbreeding had caused serious weakness in the family stock” [18, 36–39]. These instances can be taken as “character witness” testimonials that raise suspicions about the integrity of scientists. Evolution *per se* is also depicted as a tool for various atrocities, such as Nazism and Stalinism [40–43]. Oft-repeated anecdotal stories of creationist scientists being discriminated against in academy positions or peer-reviewed journals fall into the category of narrative testimonials [19, 44]. Regardless of their truth value, they are ultimately not relevant in the scientific context. Undoubtedly, science has ethical and social implications which can be discussed *per se* [45] but they are not valid evidence against scientific theories.

Isolated scientific results can also appear as testimonials. A typical creationist article introduces a scientific finding that derives from a peer-reviewed journal and re-interprets it as proof against evolution. In this manner, Doyle [46] dismisses the dinosaur–bird connection by claiming that the protofeathers of *Sinosauropteryx* are only structural collagen—a competing interpretation also among paleontologists. Doyle generalizes this as “fatal blows […] on a widely-held evolutionary idea […] these well preserved fossils prove to be wonderfully consistent with rapid burial in the global Flood”. An oft-repeated creationist attempt to refute evolution is based on the comparison of DNA or protein sequences among taxa [36, 47–48]. By misinterpreting the notion that the sequences of all eukaryotes would differ from prokaryotes by approximately the same percentage, creationists testify that “The chicken should be more advanced than the frog, the carp and especially the lamprey, and the kangaroo more advanced than all these […] This result could not be predicted by evolutionary theory” [47]. As a final example, the “dino blood” claim—findings of heme compounds interpreted as red blood cells—has become popular among creationists during the last decade [21]. Similar alleged findings of dinosaur soft tissues are considered “a deadly blow to evolutionary theory” [49] despite scientific refutations regarding preserved cells and tissues in dinosaurs [50] (see also S1 Table).

Testimonials presented as citations can take significant portions of a creationist text. When explaining why evolution is a theory in crisis, an author [47] lists several alleged flaws as follows: there is no evidence, no evolution is taking place, there are no new species, no mechanism for evolution is known, the fossil record does not support evolution, the similarity of organisms proves nothing, *etc*. Instead of evidence, these claims are backed by multiple (16) quotes. The same occurs with Davis and Kenyon [48] when they discuss the supposed lack of transitional forms in the fossil record. The issue is not researched but taken to be true by testimonials. Quotes can become the most integral part of a creationist text and even the actual conclusions consist mostly of citations when Bergman [40] presents alleged evidence that the Holocaust was a result of evolutionary theory.

### Confirmation bias

We observed aspects of confirmation bias in 100% of the sampled YEC and ID/OEC texts (Fig. 1). It includes seeking information that is consistent with existing beliefs and underutilizing or dismissing negative instances or null information [51]. Confirmation bias also occurs in science and it can arise from the positive test strategy, *i*.*e*., searching exclusively for events or data that support the hypothesis [52]. Reasons for this include “mental contamination”, in which a well-learned theory of the world can affect a person’s acceptance of incoming data and its interpretation. In addition, the bias can be very strong especially if a person should be asked to reconsider his/her position that has been “publically endorsed in the past” [51, 53]. This has also been called “myside bias”; it is common in people with diverse backgrounds and does not exclude high intelligence [54].

The cytochrome c claim offers an example to analyze confirmation bias. Creationists assume that—if evolutionary theory were true—the DNA (and protein) sequences of modern organisms should be arranged in a linear pattern that would reflect their ancestry [48]. Creationists confuse this arrangement with the pattern that would emerge from the non-available sequences of the extinct ancestors and ignore the fact that the existing sequences derive from modern organisms. Actually, present species have all evolved exactly the same time since their evolutionary paths diverged from one another and no organism is primitive in the way that its genome would have become stagnant at the point of divergence [55]. The same can be observed also in the case of the human–chimpanzee >30% difference [21], where creationists have searched confirmation to their original hypothesis (humans and apes were created separately [32]). They put emphasis on the >30% value that is observed in the Y chromosome [56] but not in the whole genome. Contradictory data (other parts of the genome show 98–99% similarity) are ignored or dismissed: “This is evidence that humans and chimpanzees are very different” [57]. “Most of their [evolutionists’] findings do not fit well with the often-repeated erroneous statement that humans and chimps are 98 percent similar, nor with the more general hypothesis that they share a common ancestor” [58].

### Pseudodiagnosticity

Pseudodiagnostics was present in 89.7% of the sampled YEC texts and in 75.0% of the ID/OEC texts (Fig. 1). This is a form of cognitive heuristics that works by regarding “presented information as relevant, regardless of its actual relevance” [12]. It can lead to the situation, where “people are insensitive […] that data may support hypotheses other than the focal hypothesis”. An example is a claim on the comparative anatomy of facial muscles in humans and other primates [49]. The claim is most often formulated as follows: The human face has 50 facial muscles (far more than apes) and the unique ability to make something like 10,000 different facial expressions [59–62]. This cannot be explained by evolutionary forces. Reinikainen [62] elaborates this by stating that “Gorillas have one half fewer facial muscles”. The first part of the claim (humans have 50 muscles) is more or less correct depending on the inclusion of different muscle groups [63]. Usually only half of the symmetrical muscles are calculated and the number is 24. The source of the next part of the claim (apes have far fewer or 50% fewer facial muscles) is hard to trace, as it is unreferenced in the above examples. Several peer-reviewed articles compare the human musculature to other primates and discuss plausible evolutionary paths between these taxa [64–66]. The second part of the claim is not supported by the literature as the number of facial muscles in chimpanzees and gorillas is 22–24. A possible explanation to the misinterpretation could be that the human facial muscles are counted by including both the left and right sides but, regarding apes, only one half of the face is included. The difference is actually very small and irrelevant when trying to disprove evolution.

Ignoring the base rate (prior probability of an event/issue being correct) occurs when people dismiss a hypothesis that would be favored by the base rate and select other hypothesis based on skewed or insufficient data [24]. A common creationist claim is that radiometric dating methods are unreliable based on few selected cases of contradictory results [67]. The base rate that consists of reproducible and robust results is ignored, although the majority of data supports the alternative hypothesis that the age of the earth has been verified to be billions of years [68]. Similar dismissal of prevailing information takes place when creationists discuss the fossil record doubting the presence of any transitional forms. Puolimatka [19] states that there would be no transitional forms in the fossil record, but the actual transitional forms are left unmentioned and the hypothesis of special creation supported. Once again, these claims are backed by testimonials from evolutionary texts [69–70]. Regarding age determination, Swenson [67] brings forth eyewitness reports: “[…] radioisotope dating […] contradicts the clear eyewitness chronology of the Word of God”.

### Re-organizing and simplifying complex information

Stereotypical thinking patterns and simplifying information were observed in 87.5 (ID/OEC)–96.6% (YEC) of the sampled creationist texts (Fig. 1). The way that complex data are re-organized into a controllable form is, again, exemplified in the cytochrome c claim. The basic finding is that when compared to humans, the cytochrome c sequences differ by an increasing degree as follows: primates < other mammals < birds and reptiles < amphibians < fish < lampreys < plants < prokaryotes [71]. In creationist texts, the results become confusing when looking at the differences from the prokaryote point of view [47]. In this case, all eukaryotes differ by approximately 65%. Creationists re-organize these data as follows:

1. Fish, amphibians, *etc*., are [according to Darwinists] ancestors of humans.
2. Ancestors are more primitive than we are. They should be more like the prokaryotes.
3. Ancient amphibians were our ancestors [according to Darwin]. Modern amphibians are similar to our ancestral amphibians (here the re-interpretation of the data by equivocating present-day amphibians to the ancestors leads to a flawed result).
4. Disclaim: All these species differ from bacteria by the same degree. No form is, thus, more primitive or ancestral.
5. Conclusion: the evolutionist hypothesis fails and the creationist theory prevails.

Re-organization of complex issues into simple forms also takes place when complicated issues are presented in a polarized form. For instance, complicated theories regarding abiogenesis (life arising from non-living matter) are presented as a dichotomy: “The RNA world did not resolve this problem. Thus, only creation is left as an option” [21]. Similar dichotomies (false dilemmas that ignore other possible alternatives in complex issues and require a choice between only two alternatives) are associated to moral issues assumedly caused by the acceptance of evolution. “If people are created in the image of God, they have to be treated accordingly […] If there is no Creator, everybody is free to do whatever he feels according to his own discretion” [47]. These are also examples of stereotypical thinking as an aspect of experiential thought patterns.

### Magical thinking, attaching moral labels and resistance to change

Moral associations were present in 62.5% of the sampled ID/OEC texts and in 93.1% of the YEC texts (Fig. 1). An example of moral/magical thinking is the creationist approach to the concept “selfish genes” [72]. While creationists sometimes acknowledge that Dawkins separated the everyday meaning of “selfish” from the evolutionary concept, the equivocation of emotional selfishness to genetic theory appears repeatedly. Creationists refuse to treat the selfish gene theory as morally neutral but associate it to teleological thinking as if genes carried out tasks due to deliberate design (see also [73] for the teleological argument for the existence of God). For instance, Puolimatka [19] claims that Dawkins gives genes “characteristics that make genes divine in some sense. They are more powerful than people, because they can manipulate people. People exist for the genes. Genes are almost eternal.”

The most striking way of attaching moral labels takes place in the association of evolutionary theory or evolutionists to the increase in abortions [41, 44, 74], to sexual minorities or “sodomy, fornication, adultery” [44] and to eugenics [19, 32]. There are also several associations of evolutionary theory to genocide, Nazism and Stalinism as well as character-assassination of evolutionary theorists, especially Darwin [18, 75]. Examples of creationist claims that have appeared repeatedly for decades despite their irrelevance to the actual scientific proof of evolution include the above-mentioned alleged moral implications of evolution (*ibid*.). Another example is the use of quotes by evolutionary proponents and Darwin repeatedly (Table 3) although their out-of-context nature is exposed. In addition, the alleged scarcity of transitional forms [19] is repeated *ad nauseam* despite scientific evidence of the opposite [76].

### Argumentative fallacies associated with experiential thinking

Our method of analyzing creationist claims makes it possible to connect argumentative fallacies [14] with the experiential thinking patterns. The types of proof that experiential thinking utilizes are *per se* fallacious from the points of view of rational logic and science (Table 4). The testimonials regarding dismissal of evolution do not disproof actual scientific data. Nor are the characteristics of scientists or alleged consequences of evolutionary theory relevant. Thus, these testimonials can be classified as appeals to authority similar to the multitude of quotes. Out-of-context citations (Table 3) allegedly affirming dishonesty or fatal flaws in evolutionary theory can be regarded as quote mining and *tu quoque* fallacies. Testimonials containing moral issues belong to the fallacies of guilt by association and direct *ad hominem* and testimonials about the personal disbelief about evolutionary theory or its “irrationality” are examples of the argument from incredulity fallacy [72].

**Table 4:** Characteristics of experiential thinking in creationist texts and their comparison with fallacies.

Characteristic	Form	Argumentative fallacies
Testimonial	“Hard to believe in evolution”, “evolution not based on evidence”	Appeal to incredulity, *ad ignorantiam*
	“A scientist converted to theism because of problems in evolution”	Appeal to authority
	Character witness: demonization of evolutionists	*Ad hominem*
	Character witness: famous scientists were Christians	Appeal to authority
	Scientists tell that they have been discriminated if they are creationists	Appeal to pity, appeal to consequences
	Citing evolutionists as admitting weaknesses in evolutionary evidence	*Tu quoque*, quote mining, appeal to authority
	Scientists telling they would not accept supernatural explanations in any case	Poisoning the well, quote mining
	Evolutionary theory leads to atrocities, *e*.*g*., immorality, Nazism, Stalinism, eugenics, genocide	Guilt by association, *ad consequentiam*, slippery slope
	Holocaust survivors testify that Darwinism was a causative agent in atrocities	Guilt by association, *ad consequentiam*, appeal to authority, appeal to pity
	Selecting particular scientific data/articles out of context as evidence for creationism	Hasty generalization, straw man
Confirmation bias	Ignoring negative/contradictory information	Hasty generalization
	Re-organizing complex data into a simple form	Straw man, false dilemma
	Ignoring base rate	Hasty generalization
Attaching moral labels	Demonizing evolutionary proponents as atheists, unreliable, unqualified, *etc*.	*Ad hominem*
	Evolutionary theory associated to Nazism, Stalinism, *etc*.	Guilt by association, *ad consequentiam*, slippery slope
	“Evolutionists themselves make religious arguments”	*Tu quoque*, equivocation
	“Not to believe in Biblical inerrancy brings 'grave consequences', both to the individual and to the Church”	Appeal to fear and force (*ad baculum*)

Claims based on confirmation bias, ignoring contradictory data and base rates or re-organization of complex data lead to hasty generalizations and straw men. The stereotypical re-organization of complicated data into dichotomies is an example of a false dilemma. Together the testimonials and biased quoting or data selection provide the creationist audience with emotionally appealing “evidence” that not only informs about personal experience of the author [12] but also directs the creationist author to use fallacies as arguments. The fallacies form a background upon which the “scientific” claims are placed. Thus, addressing fallacious arguments is not only quibbling about logical mistakes. On the contrary, fallacies can be active tools in persuasion and create or enforce false beliefs [13]. The same is true of experiential thinking patterns and erroneous conceptions of science [12]. Thus, both experiential thinking and the resulting fallacious argumentation are not irrelevant when discussing the understanding and acceptance of evolutionary theory.

### Prevalence of aspects of experiential thinking in pro-evolutionary texts

The sampled pro-evolutionary texts contained a high prevalence of testimonials (93.3%) and moral associations (80.0%) but lower prevalences of stereotypical thinking (46.7%), confirmation bias (26.7%) and pseudodiagnostics (6.7%) than the YEC and ID/OEC writings (p < 0.001). Testimonials included personal support to TE instead of creationism, such as “I hold an alternate view of reality which integrates God’s revelation in His Word and in His works” [77]. Demonization against the qualifications of creationists also appeared: “I can only conclude that he’s [a creationist] become so convinced his actual Christian salvation is bound to believing this dreck he can’t bring himself to read the actual research” [78] and labeling a supporter of creationism “a raving idiot” [79]. There were also examples of generalization and disqualification of creationists [80], for instance, it was stated that “[…] the claims made by creationists show that almost all of them are woefully ignorant of evolution” [81].

Moral issues associated to pro-evolutionary refutations of creationist claims were present in the form of character assassination: “deplorable deceiver […] a sad end for a world-class researcher” [82]. Generally, the creationist worldview was associated to atrocities: ”The truly appalling thing all such people [fundamentalists] have in common, whether they are incited to murder by ayatollahs or to less violent observances by television evangelists […]” [83]. When connecting moral issues to the evolutionist–creationist debate, testimonials in the form of quotations also appeared similar to the out-of-context citations in the creationist sample material [84–87]. Appeals to historical figures (*e*.*g*., Hitler) were also employed to demonize creationists [88]. Finally, accusations for dishonesty were counter-attacked in a similar manner by moral arguments in pro-evolutionary texts [89–91].

## Discussion

### Prevalence of experiential thinking

The high prevalence of the analyzed aspects of experiential thinking does not mean that all creationist thought would be irrational, nor is it possible to state based on the analysis that YEC or ID/OEC claims *per se* would be flawed—that is to be determined by actual scientific evidence. The results still reveal that these claims are often supported by testimonials and moral arguments, they derive from material that can be skewed (confirmation bias, dismissal of negative data) and there is a lack of alternative hypotheses, even when an overwhelming majority of data would not support the creationist case.

Reciprocally, scientists should be conscious of the aspects of experiential thinking in anti-creationism rebuttals. Stereotypical and biased conceptions of creationist theory can lead to straw man argumentation. This includes misconceptions, such as claims that creationists would believe in the fixity of biological species (in fact, they refer to “kinds”) and that creationists would be opposed to science *per se* [15]. Our results suggest that, although the actual evolutionary science would not be based on experiential thinking, the creationist–evolutionary debate contains non-scientific aspects on both sides.

Regarding the English- and Finnish-language texts, there were no statistical differences in the use of testimonials or any other aspects of experiential thinking in the sampled creationist texts. Also the quotes of scientists used to promote creationism in Finland were often direct translations of the English original. This emphasizes the international nature of creationist claims as noted also by Numbers [15].

### Is experiential thinking unscientific?

An alternative hypothesis to explain the findings in the sample material is the possibility that creationist authors do not exhibit experiential thinking but the citations and selected data would include rational scientific deduction. It must be assessed if the findings interpreted as testimonials could be valid scientific evidence against evolutionary theory. While the quotations can *per se* be *verbatim* correct and derive from the alleged sources (although sometimes out-of-context; Table 3), it should be the actual observations and not the person that matter. In addition, confirmation bias and the apparent dismissal of negative data, such as peer-reviewed articles that do not support the creationist case, yield additional evidence for the presence of experiential thinking. The disregard for baseline data was also very obvious. An example of this is the creationist habit of dismissing radiometric dating procedures based on selected discrepancies [47, 67, 92].

It has been suggested that confirmation bias in the form of commitments should not be automatically excluded from scientific thinking [93]. Generally, scientists accept that they ought to be emotionally attached to their hypotheses and not to exclude the benefits of strong, prior commitments. It could be argued that in the field of creationism, these prior commitments to supernatural hypotheses could be analogous to the commitments of biologists [19, 36, 47, 74, 94]. In this respect, it is important to remember that quoted testimonials and personal observations or experience can belong to the scientific method. They can provide research ideas and hypotheses to be studied further. Research can eventually yield rigorous evidence to support the original experience or testimonial to become accepted as plausible theories. This requires experimentation and observations and acceptance of negative data, which is where the creationist case has failed to deliver. It has also been suggested that the confirmation bias present in the psychological profile of some religious persons can ensure that even a small number of positive feedback can outweigh the failures and produce conviction of, *e*.*g*., supernatural interventions [95]. We suggest that commitment to one’s hypotheses is acceptable for scientists and creationists, but hypotheses should be subjected to criticism and alternative hypotheses should be sought actively without dismissing them with confirmation bias.

In the sampled creationist texts, there was a failure to consider alternative hypotheses (*e*.*g*., human–chimpanzee genetic difference claim), null information (cytochrome c and ancestors claim) and negative evidence (facial muscle claim). Regarding this issue, we suggest that the creationist hypotheses should be given a thorough scientific assessment but, of course, this has mostly been done several years ago [16, 96]. It should be adequate to review and criticize each creationist hypothesis in detail only once, if no additional evidence is provided. Moreover, scientific rebuttals should be realized without resorting to counter-fallacies and without getting involved in debates that are not related to evolutionary theory *per se* (such as the claims of evolutionary theory leading to Nazism). The present analysis supplements the scientific assessment of creationist claims and suggests that one reason for the apparent disregard of scientific refutations could be the reliance on experiential thinking and fallacies which are not adequately addressed in scientific responses.

The results also show that creationism has a very strong tendency to give evolutionary theory moral significance. Actions of the actual people committing atrocities are almost dismissed in the creationist rhetoric, as evolutionary theory or “Darwinism” *per se* is held responsible for immorality, eugenics, abortions, Nazism, Stalinism and eventually genocide [18–19, 40]. This leads to the fallacies of guilt by association, appeal to consequences and the slippery slope argument exemplifying the moral attachment of experiential thinking but having no value in proving creationist theory. Regarding the attachment of undesirable moral characteristics to evolutionary theory proponents, different *ad hominem* fallacies such as the direct *ad hominem* in the form of demonization are also prevalent [97].

Creationist authors have criticized the use of “demarcation criteria”, *i*.*e*., exclusion of supernatural explanations from evolutionary theory by professionals in natural sciences, while at the same time discussing the characteristics of the “designer” in the context of ID/OEC [19]. We present here a hypothetical demarcation line, the division of explanations on observable phenomena into scientific thinking and into interpretations based on experiential thinking. In the case of creationists, the development of biodiversity on the earth seemingly rests at least partly on the experiential side. While it is certainly plausible that practically everybody uses experiential thinking in various everyday activities [12, 98], natural scientists use rational methods for acquiring evidence to test scientific hypotheses. Based on the sample material, scientific rebuttals also contained many instances of experiential thinking, including testimonials and moral implications, although it must be emphasized that the sample material did not include peer-reviewed journal papers but texts debating creationism and evolution. We can also surmise that a part of the population and even scientists who “believe” in evolution could also process evolutionary theory based on experiential thinking and not consider alternative hypotheses. In addition, it is not impossible that one person can endorse in a seemingly irrational manner both creationism and evolution [3]. It is important for scientists to remember that while theories can be taught as truths, there is always the possibility of revising these “truths” with new evidence [99].

### Experiential thinking and fallacies, and a scheme for analyzing creationist texts systematically

The present analysis offers a link between creationist thinking patterns and the abundance of argumentative fallacies in creationist texts. Emotional but scientifically irrelevant fallacies—demonization of scientists, association of evolutionary theory to atrocities, appeals to authority, hasty generalizations and straw man fallacies—are typical of creationist writings. This habit of resorting to fallacies becomes understandable if we consider what experiential thinking judges to be the most convincing evidence: personal testimony and emotional commitment [12]. Using this type of “evidence” contains by definition personal opinions, citations and descriptions of personal characteristics instead of actual observational or experimental data. This includes appeals to authority and acting like a character witness. These arguments use moral characteristics of scientists (or a theory) instead of scientific observations, which can lead to various forms of *ad hominem* and appeals to consequences. These are regarded as fallacious if we consider their scientific content in proving or disproving evolutionary theory. In experiential thinking, however, the fallacious arguments become the actual evidence.

A unified theory on creationist thinking is starting to emerge from the combination of analyses regarding experiential thinking and fallacious arguments. The sampled creationist writings revealed several aspects of experiential thinking including testimonials, narratives, confirmation bias, pseudodiagnostics and applying morality to data that do not concern ethics. Alternative hypotheses for the observed experiential thinking in creationist texts could be considered unlikely. Characteristic of experiential thinking, creationist evidence consists especially of quotes, appeals to authority and other types of testimonials that are chosen by studying scientific information through pseudodiagnostics and confirmation bias. The evidence that has been gathered and interpreted by scientists with rational methods is subjugated to experiential thinking, and evolutionary theory is being criticized with testimonials and biased data. This can be contradictory by itself: it is not possible to disprove a theory based on rational observations with experiential evidence. Instead, actual data should be gathered, analyzed, interpreted and compared to previous knowledge.

The utilization of experiential thinking results in argumentative fallacies. In the world of science, experiences and testimonials are not direct evidence but they can become testable hypotheses. When argumentation analysis is applied, testimonials become appeals to authority, *ad hominem*, poisoning the well and guilt by association fallacies and appeals to ignorance and incredulity. Is this connection between experiential thinking and the reliance on emotional fallacies causal? While the present analysis cannot answer this, the similarities of experiential thinking and fallacies in argumentation theory are so striking that they could possibly be interrelated. This could be one of the causes for the big discrepancy between creationists and evolutionary proponents that is hard to reconcile.

The persistence of the same creationist claims for several decades is also an aspect of experiential thinking and indicates that the strategy of discussing the scientific aspects while also resorting to counter-fallacies by scientists and educators has been unproductive. An alternative approach would be to address not only the scientific misinterpretations but also the thinking patterns and argumentation. These thinking patterns and argumentation should be recognized and addressed but not rebutted with emotional counter-fallacies and reciprocal utilization of experiential thinking. This offers a possible exit from the stalemate and the vicious circle of fallacies and counter-fallacies. A proposed scheme can be summarized as follows (see also S1 Table):

- Examine the claim by source criticism and assess if it is a correct portrayal of the original scientific data.
- If the source is not cited correctly, assess if the errors have been addressed.
- Recognize if there are signs of experiential thinking.
- Examine if argumentative fallacies are present.
- Examine if experiential thinking and fallacies appear in context with scientific issues.
- Summarize the findings.

While irrelevant as scientific proofs, aspects of experiential thinking and fallacies can be emotionally relevant to persons of both creationist and evolutionary worldviews. They can also be relevant topics of discussion and research, even if they are not evolutionary science. When addressing students with creationist convictions or backgrounds, these topics can be discussed in a respectful manner separate from the actual scientific issues. The aim of the classroom discussion need not be to convert anyone. The assessment of fallacies and experiential thinking patterns can, however, unravel if some of the perceived contradictions are not actually related to natural sciences but, *e*.*g*., ethics (moral dimensions of evolutionary debate). Obviously, the outcome would not necessarily be a consensus, and many issues such as the age of the earth can remain disputed, but by focusing on scientific issues the debates could become more constructive. The method can also be of equal benefit for creationists, if their aim is to engage in an unprejudiced scientific discussion. The systematic analysis of experiential thinking patterns and fallacies can tell the YEC or ID proponent why his/her arguments are not always “taken seriously” in the scientific community and how to extract the actual science from the background to make one’s case more presentable. In addition, the presence of experiential thinking and fallacies in pro-evolutionary texts can be a factor in their dismissal by creationists and contribute to the resistance to change observed in the debate.

## Supporting Information

S1 Table Proposed scheme and example how to address aspects of experiential thinking and argumentative fallacies in creationist claims in table format. (PDF)

S1 Table Original data on the texts included in the statistical analyses. (PDF)
