# Correction: Experiential Thinking in Creationism—A Textual Analysis

**DOI:** 10.1371/journal.pone.0123488

**Published:** 2015-04-01

**Authors:** The PLOS ONE Staff

Fig. 1 is incorrectly missing asterisks denoting statistical significance. The authors have provided a corrected version of Fig. 1 here.

**Fig 1:**
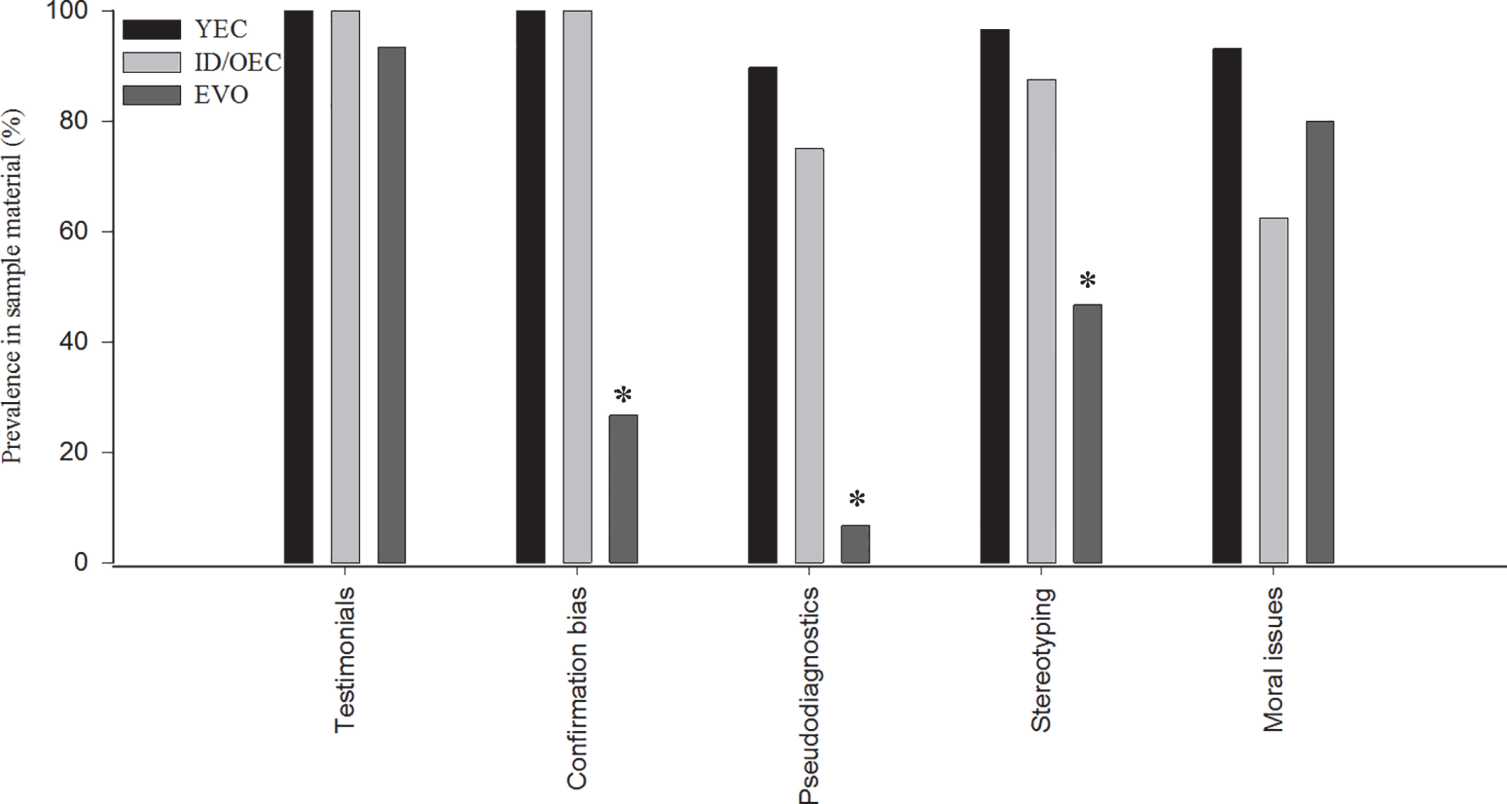
Prevalences (%) of selected aspects of experiential thinking in the sampled material representing young-earth creationism (YEC; n = 29), intelligent design/old-earth creationism (ID/OEC; n = 8) and pro-evolutionary texts (EVO; n = 15). “Testimonials” include personal testimonies, quotes, appeals to authorities, *etc*. “Confirmation bias” represents ignoring or dismissing contradictory data and alternative hypotheses. “Pseudodiagnostics” entails giving high relevance to misinterpreted or irrelevant issues. “Stereotyping” includes dichotomies and generalizations and “moral issues” refer to scientifically irrelevant discussion of moral implications to prove or disprove a claim. * = Difference between the text types (χ^2^-test, Fisher’s exact test, p < 0.001).n/old-earth creationism (ID/OEC; n = 8) and pro-evolutionary texts (EVO; n = 15).
