# Supplementary material for: Experiential Thinking in Creationism—A Textual Analysis
Source: PLoS One. 2015 Mar 3;10(3):e0118314. doi: 10.1371/journal.pone.0118314 (PMC4348421; doi:10.1371/journal.pone.0118314)
Supplement: S1 Table — (PDF) [file pone.0118314.s001.pdf]

S1 Table. Proposed scheme and example how to address aspects of experiential thinking and argumentative fallacies in creationist claims. The example is the claim that there are findings of dinosaur bones with blood vessels and blood and bone cells [1-6] allegedly discovered by Schweitzer et al. [7-8].

**a. Source criticism: is the claim a correct portrayal of the original data?**<br>• No. The original articles describe the alleged red blood cells as heme-containing compounds and/or hemoglobin breakdown products and the bone cells as cell-like microstructures. In addition, later studies suggest that the alleged flexible vascular tissues and osteocytes in demineralized bone could represent bacterial biofilms [9].
**b. If the source is not cited correctly, has this been addressed?**<br>• Yes, on multiple occasions [10] including also religiously motivated texts [11-13].<br>• Despite rebuttals the claim persists.
**c. Are aspects of experiential thinking present?**<br>• Yes, as follows:<br>1. Testimonials: citations of a popularized paper [14] used to prove the claim instead of careful explanations about the original peer-reviewed articles.<br>2. Confirmation bias: information not supporting the creationist claim is dismissed (*e.g.*, heme compounds not blood, other data on the timing of the era of dinosaurs are dismissed).<br>3. Pseudodiagnostics: erroneously cited data taken as pivotal when considering the validity of evolutionary theory *per se*, while, in fact, the original articles do not concern evolution. Is base rate ignored? Yes, if dinosaurs had lived during historical era, findings of soft tissues would be expected to be frequent.<br>4. Is there a tendency for broad generalization and/or stereotypical thinking? Yes, this finding allegedly makes evolutionary theory false. Hemoglobin-related substances (heme) are interpreted as red blood cells.<br>5. Is moral significance attached to the data? Yes, the scientist's integrity and religiosity are questioned.
**d. Are there argumentative fallacies?**<br>• Yes, as follows:<br>1. Testimonials leading to appeals to authority (citations of scientists instead of evidence).<br>2. Moral issues leading to *ad hominem* (questioning the integrity of the scientist), for instance by Reinikainen [3]: "[The scientist], having been forced under a lot of pressure […] has tried to wriggle out of these observations […] to preserve her credibility in the scientific community."<br>3. Confirmation bias leading to hasty generalizations ("a deadly blow to evolutionary theory" [15]).<br>4. Confirmation bias and pseudodiagnostics leading to equivocations (heme compounds = blood).
**e. Do the fallacies and aspects of experiential thinking appear in the context of scientific issues, thus, potentially forming a biased background for the audience to evaluate the claim?**<br>• Yes, the claim is presented in a fallacious context and the fallacies and the narrative related to experiential thinking appeal to emotions and can either create or enforce false beliefs.
**f. Recognize and address not only the scientific claim but also the findings of experiential thinking and fallacies and bring up the possibility of the fallacious context creating false beliefs and making them persuasive. Avoid counter-fallacies. Summarize your findings, for example, as follows:**<br>• The claim cites the original papers erroneously and it has been repeatedly rebutted. The claim rests on experiential thinking patterns (testimonials, moralization, generalizations and ignoring negative and contradictory data and base rates). The claim is also accompanied with argumentative fallacies including *ad hominem*, hasty generalizations and equivocations. This context of experiential thinking and fallacies can be important for the acceptance of the claim as the context creates and enforces false beliefs. The unscientific aspects can, however, be emotionally important for students and educators and provide interesting discussion topics while acknowledging their irrelevance as proofs for or against evolutionary theory.

1. Reinikainen P. Darwin vai älykäs suunnitelma [Darwin or intelligent design]. Helsinki: Uusi Tie; 2011. In Finnish.
2. Reinikainen P. Enkeleitä alemmaksi [Lower than angels]. 2013 [cited 15 December 2013]. In: Pekkareinikainen.info [Internet]. Available: http://www.pekkareinikainen.info/fi/index.php?option=com_content&task=view&id=24&Itemid=27. Webpage removed in 2014. In Finnish.
3. Reinikainen P. Dinosaurusten arvoitus ja Raamattu [The enigma of the dinosaurs and the Bible]. 2nd ed. Helsinki: Kuva ja Sana; 2003. In Finnish.
4. Wieland C. Sensational dinosaur blood report! Creation. 1997;19: 42-43.
5. Catchpoole D, Sarfati J. 'Schweitzer's dangerous discovery'. 2006 July 19 [cited 17 May 2014]. In: Creation.com [Internet]. Eight Mile Plains: Creation Ministries International. Available: http://creation.com/schweitzers-dangerous-discovery.
6. Answers in Genesis. The scrambling continues: an update on the amazing T. rex bone discovery announced a year ago this month. 2006 March 6 [cited 17 November 2014]. In: Answersingenesis.org [Internet]. Hebron: Answers in Genesis. Available: https://answersingenesis.org/dinosaurs/bones/the-scrambling-continues/.

7. Schweitzer MH, Marshall M, Carron K, Bohle DS, Busse SC, Arnold EV, et al. Heme compounds in dinosaur trabecular bone. Proc Natl Acad Sci. 1997;94: 6291-6296.
8. Schweitzer MH, Wittmeyer JL, Horner JR, Toporski JK. Soft-tissue vessels and cellular preservation in *Tyrannosaurus rex*. Science. 2005;307: 1952-1955.
9. Kaye TG, Gaugler G, Sawlowicz Z. Dinosaurian soft tissues interpreted as bacterial biofilms. PLoS ONE. 2008;3: e2808**.**
10. TalkOrigins archive. Claim CC371. 2003 Nov 7 [cited 17 November 2014]. In: Talkorigins.org [Internet]. Available: http://www.talkorigins.org/indexcc/CC/CC371.html.
11. Moore G. Dinosaur blood? 2011 August 1 [cited 17 May 2014]. In: Reasons.org [Internet]. Covina: Reasons to Believe. Available: http://www.reasons.org/articles/dinosaur-blood.
12. Moore G. Dinosaur blood revisited, part 1 (of 2). 2011 August 3 [cited 17 May 2014]. In: Reasons.org [Internet]. Covina: Reasons to Believe. Available: http://www.reasons.org/articles/dinosaur-blood-revisited-part-1-of-2.
13. Moore G. Dinosaur blood revisited, part 2 (of 2). 2011 August 6 [cited 17 May 2014]. In: Reasons.org [Internet]. Covina: Reasons to Believe. Available: http://www.reasons.org/articles/dinosaur-blood-revisited-part-2-of-2.
14. Schweitzer M, Staedter T. The real Jurassic park. Earth. 1997;6: 54.
15. Reinikainen P. Evoluutio vai luominen? [Evolution or creation?]. 2013 [cited 15 December 2013]. In: Pekkareinikainen.info [Internet]. Available: http://www.pekkareinikainen.info/fi/index.php?option=com_content&task=view&id=25&Itemid=27. Webpage removed in 2014. In Finnish.
