# Supplementary material for: Experiential Thinking in Creationism—A Textual Analysis
Source: PLoS One. 2015 Mar 3;10(3):e0118314. doi: 10.1371/journal.pone.0118314 (PMC4348421; doi:10.1371/journal.pone.0118314)
Supplement: S1 Table — (PDF) [file pone.0118314.s002.pdf]

S2 Table. Original data on the texts included in the statistical analyses.

Type	Language	Testimonials	Confirmation bias	Pseudodiagnostics	Stereotypical thinking	Moral association
1	1	1	1	1	1	1
1	1	1	1	1	0	1
1	1	1	1	1	1	1
1	1	1	1	1	1	1
1	1	1	1	1	1	1
1	1	1	1	1	1	1
1	1	1	1	1	1	1
1	1	1	1	1	1	1
1	1	1	1	1	1	1
1	1	1	1	1	1	1
1	1	1	1	1	1	1
1	1	1	1	1	1	1
1	1	1	1	1	1	1
1	1	1	1	1	1	1
1	1	1	1	1	1	0
1	1	1	1	0	1	1
1	2	1	1	1	1	1
1	1	1	1	0	1	1
1	1	1	1	1	1	1
1	1	1	1	1	1	1
1	1	1	1	1	1	1
1	2	1	1	1	1	1
1	2	1	1	1	1	1
1	2	1	1	1	1	1
1	2	1	1	1	1	1
1	2	1	1	1	1	1
1	1	1	1	0	1	1
1	1	1	1	1	1	0
1	1	1	1	1	1	1
2	1	1	1	0	0	0
2	1	1	1	1	1	0
2	1	1	1	1	1	1
2	1	1	1	1	1	1
2	1	1	1	0	1	1
2	1	1	1	1	1	0
2	2	1	1	1	1	1
2	2	1	1	1	1	1
3	1	1	1	0	1	1
3	1	1	0	0	0	1
3	1	1	0	0	1	1
3	1	1	0	0	1	1
3	1	1	1	1	1	1
3	1	1	0	0	1	1
3	1	1	0	0	0	1
3	1	1	0	0	0	1
3	1	1	0	0	0	1
3	1	1	1	0	1	1
3	1	1	0	0	0	0
3	1	0	1	0	1	0
3	1	1	0	0	0	1
3	1	1	0	0	0	0
3	1	1	0	0	0	1
1=YEC	1=English	0=absent	0=absent	0=absent	0=absent	0=absent
2=ID/OEC	2=Finnish	1=present	1=present	1=present	1=present	1=present
3=Pro-evolutionary						
